# A feasibility study examining the effect on lung cancer diagnosis of offering a chest X-ray to higher-risk patients with chest symptoms: protocol for a randomized controlled trial

**DOI:** 10.1186/1745-6215-14-405

**Published:** 2013-11-26

**Authors:** Christopher N Hurt, Kirsty Roberts, Trevor K Rogers, Gareth O Griffiths, Kerry Hood, Hayley Prout, Annmarie Nelson, Jim Fitzgibbon, Allan Barham, Emma Thomas-Jones, Rhiannon Tudor Edwards, Seow Tien Yeo, William Hamilton, Angela Tod, Richard D Neal

**Affiliations:** 1 Wales Cancer Trials Unit, School of Medicine, Cardiff University, Cardiff, UK; 2 Doncaster Royal Infirmary, Doncaster and Bassetlaw NHS Foundation Trust, Doncaster, UK; 3 South East Wales Cancer Trials Unit, School of Medicine, Cardiff University, Cardiff, UK; 4 Marie Curie Palliative Care Research Centre School of Medicine, Cardiff University, Cardiff, UK; 5 Patient representatives, Cardiff, UK; 6 Centre for Health Economics and Medicines Evaluation, Bangor University, Gwynedd, UK; 7 University of Exeter Medical School, Exeter, UK; 8 Centre for Health and Social Care Research, Sheffield Hallam University, Sheffield, UK; 9 North Wales Centre for Primary Care Research, Bangor University, Wrexham, UK

## Abstract

**Background:**

In order to improve lung cancer survival in the UK, a greater proportion of resectable cancers must be diagnosed. It is likely that resectability rates would be increased by more timely diagnosis. Aside from screening, the only way of achieving this is to reduce the time to diagnosis in symptomatic cancers. Currently, lung cancers are mainly diagnosed by general practitioners (GPs) using the National Institute for Health and Clinical Excellence (NICE) guidelines for urgent referral for chest X-ray, which recommend urgent imaging or referral for patients who have one of a number of chest symptoms for more than 3 weeks. We are proposing to expand this recommendation to include one of a number of chest symptoms of any duration in higher-risk patients.

**Methods/Design:**

We intend to conduct a trial of imaging in these higher-risk patients and compare it with NICE guidelines to see if imaging improves stage at diagnosis and resection rates. This trial would have to be large (and consequently resource-intensive) because most of these patients will not have lung cancer, making optimal design crucial. We are therefore conducting a pilot trial that will ascertain the feasibility of running a full trial and provide key information that will be required in order to design the full trial.

**Discussion:**

This trial will assess the feasibility and inform the design of a large, UK-wide, clinical trial of a change to the NICE guidelines for urgent referral for chest X-ray for suspected lung cancer. It utilizes a combination of workshop, health economic, quality of life, qualitative, and quantitative methods in order to fully assess feasibility.

**Trial registration:**

Clinicaltrials.gov NCT01344005

## Background

In 2010, lung cancer caused over 34,800 deaths in the UK, accounting for 7% of all deaths and 22% of all deaths from cancer [1,2]. Five-year survival in the UK is just over 5%, compared with 13% in the USA and similar proportions for several other EU countries [3]. Compared with other countries, patients in the UK have more advanced stage at presentation and a lower rate of lung cancer resections (10%) [3-7]. It has been estimated that 5,000 to 10,000 lives are lost annually in the UK because of probable later diagnosis of cancer [8-10].

Most commonly, lung cancer is diagnosed in the UK following symptomatic presentation to primary care [11]. NICE guidelines have identified the symptoms that should trigger referral for a chest X-ray [12], and suspicious chest X-rays should then be urgently referred to secondary care [11]. The guidelines are currently being updated.

Many patients have had symptoms for a year or more prior to diagnosis [13,14]. Most of the overall time to diagnosis is accounted for by patient or primary care delay, with referral and secondary care delays accounting for relatively less time [15], raising the hypothesis that management of symptoms by GPs, before ordering an X-ray, may delay the diagnosis [16]. A previous study identified a range of factors that delayed GP referral of patients for a chest X-ray including: symptom experience, lack of knowledge, fear, cultural factors, non-standard patterns of healthcare utilization, underlying stoical attitudes, and blame and stigma because of smoking [17]. In the UK, only 23% of diagnoses are made through urgent referrals based on NICE guidelines [18], and patients who are diagnosed following an urgent referral have more advanced TNM (tumor, node, metastasis) stage compared with patients diagnosed through other routes [19], suggesting that the guidance currently identifies those with more advanced disease. The effect of a more or less timely diagnosis is unproven in lung cancer, although there are good arguments to suggest benefit [20].

Several studies of screening with plain X-ray and sputum cytology were undertaken in the 1970s and 1980s. Although none found a mortality benefit [21-23], they all had methodological weaknesses, with the controls receiving some screening, and small sample sizes resulting in inadequate power. However, further information on the value of plain X-ray has recently been provided by the large PLCO (Prostate, Lung, Colorectal and Ovarian Cancer Screening) Trial [24], which assessed the effects of annual screening with modern chest radiography for 4 years in 77,445 subjects compared with 77,456 controls who received usual care. Although screening detected more cancers and more early-stage cancers, there was no evidence of genuine benefit, with no reduction in mortality over 13 years, either in the screened group as a whole, or in a high-risk subgroup (based on the National Lung Screening Trial (NLST) trial inclusion criteria, see below).

The advent of low-dose computed tomography (CT) provided a new tool for screening. In 2011, the landmark American NLST reported the effect of screening for 3 years of current or former smokers aged 55 to 74 with a smoking history of at least 30 pack-years: there was a 20% reduction in mortality from lung cancer and a 6% decrease in all-cause mortality [25]. However, there were serious costs incurred. Suspicious nodules requiring further evaluation were found in 20% of the participants, more than 90% of whom did not have lung cancer. Over-diagnosis, meaning the identification of indolent cancers that would never have become clinically apparent, was estimated to apply to about 25% of the cancers found [26]. It has been estimated that to implement this program nationwide using the NLST inclusion and exclusion criteria would cost $2 billion per annum in CT scan costs alone [27]. When added to the complexities of recruitment of a population that is likely to be hard to reach, and a complex interaction with smoking cessation [28], it is unsurprising that widespread uptake of screening based on this protocol has not occurred.

The largest European low-dose CT screening study, the Nederlands-Leuvens Longkanker Screenings Onderzoek (NELSON) trial, used a longer screening interval than the NSLT and a volumetric method of assessing nodules. Although it has reported a favorable cancer-stage distribution at diagnosis [29], determination of a genuine stage shift at detection, from advanced stage to early stage, will require comparison with the control group. This has not yet been reported, and the size of any benefit resulting from this method remains to be determined.

On the current evidence, we believe that the current best hope for improving outcomes in lung cancer remains with earlier recognition of symptomatic disease at presentation to primary care, and this is most readily done with a chest X-ray. This is because patients who are both high risk and symptomatic have a higher risk of lung cancer than those high-risk patients included in the aforementioned screening studies. A systematic review of interventions to reduce patient and practitioner delay in cancers did not identify any studies relating to lung cancer [30]. A systematic review of interventions to reduce primary care delay in cancer referral did not find any studies that reported a direct effect on reducing delay, or any studies in lung cancer [30].

### The intervention

**Figure 1:**
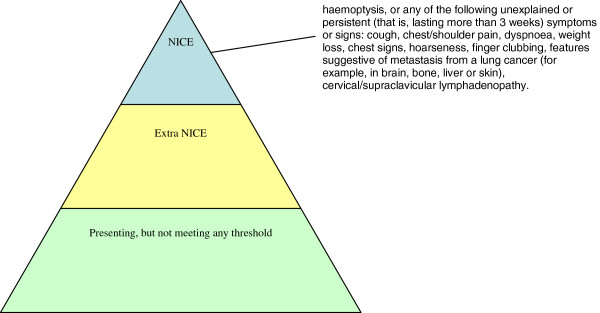
Thresholds for referral for chest X-ray.

We propose to lower the threshold (see Figure 1) for a chest X-ray for potential lung cancer symptoms in high-risk patients (that is, the new diagnostic strategy, termed ‘extra-NICE’). This will be compared with current NICE guidance (‘NICE’). Extra-NICE recommends a chest X-ray if one has not been obtained within the previous 3 months, the patient is aged over 60 years, and is a smoker or ex-smoker with 10 or more pack-years of smoking and with: a new or altered cough of any duration reported to primary care, and/or increased breathlessness or wheezing (whether or not associated with purulent sputum).

Although tumor doubling time is variable, we hypothesize that if tumors are identified earlier, it may be possible to achieve a stage shift, which may translate into higher rates of resections and other radical treatments and an overall improvement in lung cancer survival.

### Main research question

Ultimately, we want to conduct a randomized trial to determine whether or not our extra-NICE guidelines improve lung cancer survival, using resection rates and survival as outcome measures. Using resection rate, which is well correlated with survival, as the main end point of a full trial, and looking for an improvement from 10% to 15%, we would require approximately 1,900 lung cancers, necessitating a UK-wide, expensive trial. Using survival as the main endpoint would require even more cancers. This pilot trial will provide information that will inform the planning of the full trial.

This study fulfills the NETSCC (National Institute for Health Research Trials and Studies Coordinating Centre) definition of both a feasibility and pilot study because it assesses how well the components of the full trial will work together, key parameters that will be required to plan the full trial, and whether or not it is realistically achievable [31].

#### ***Clinical information***

The basic clinical information required is as follows: the prevalence of ‘extra-NICE’ symptoms in patients consulting in UK general practice, the proportion of those who agree to participate, and the proportion of those who are diagnosed with lung cancer.

Of those patients diagnosed with lung cancer, we then require: stage at diagnosis, performance status, and the proportion of patients receiving radical treatments.

#### ***Process information***

The process information required is: the feasibility of primary care recruitment and randomization of patients to a chest X-ray, or not; the best sources of routine data for capturing lung cancers; the best way to train GPs to identify and recruit eligible patients into the trial; the most effective method of presenting the trial (and randomization) to patients; barriers to recruitment and how can we overcome them; and the best measures of resource use and health-related quality of life measures to facilitate health economic analysis of the cost-effectiveness of the extra-NICE protocol.

## Methods/design

### Study design

Ethics approval for the design described here was received from the North Wales Research Ethics Committee (Reference: 11/WA/0222) and is in compliance with the Helsinki Declaration.

**Figure 2:**
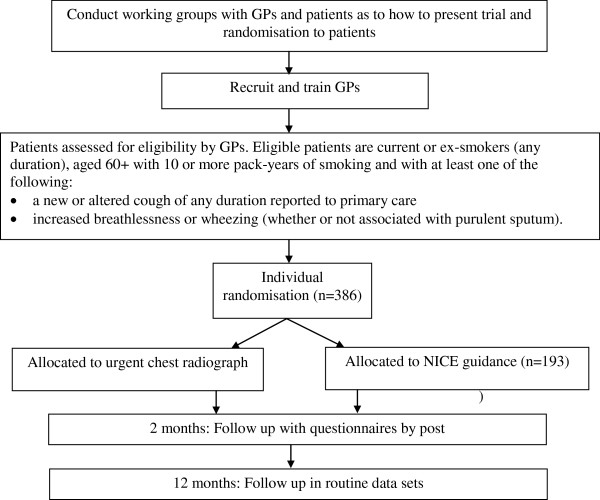
Trial schema.

The study will have two elements: a Working Group and a pilot trial (see Figure 2) with qualitative and health economic components.

The Working Group of patients and GPs will be convened to establish: the best way to train clinicians to identify and recruit eligible patients into the trial; and the most effective method of presenting the trial (and randomization) to patients (including review of Patient Information Sheet and Informed Consent form).

The chief investigator (CI) will facilitate the Working Group, which will consist of two patient representatives, two non-academic GPs, two nurse practitioners, research network representatives, the trial manager, and a qualitative researcher. Proceedings will be collated in a report highlighting key points to take forward.

We will survey all GPs in South-East Wales to assess the level of interest in the proposed full trial. For the pilot trial, we will select approximately 20 general practices in south-east Wales, and a small number from north Wales and south Yorkshire. GPs at these practices will be trained to recruit patients who fulfill the requirements for extra-NICE (but who do not meet the NICE threshold for referral). Patients willing to participate will be individually randomized 1:1 to one of two arms. Arm A will be the NICE guidance arm; the patient should be treated according to current NICE guidance. Arm B will use the extra-NICE protocol; that is, urgent referral for chest X-ray: the GP orders an urgent chest X-ray through the usual local process.

### Participant eligibility

Eligible patients will be identified within general practices, either during consultations, in chronic obstructive pulmonary disease (COPD) clinics or by retrospective searches of the records of the previous week’s consultations. To be eligible to be included in the trial the patient must meet all of the following criteria: 1) age over 60 years, 2) smoker or ex-smoker (any duration) with 10 or more pack-years of smoking (assessed using http://smokingpackyears.com/. or by dividing the number of cigarettes smoked per day by 20 and multiplying by the number of years smoked), and 3) has given written informed consent; and at least one of the following criteria: 1) a new or altered cough of any duration reported to primary care or 2) increased breathlessness or wheezing (whether or not associated with purulent sputum).

None of the following categories of patients can be included in the trial: 1) patients who qualify for urgent referral for chest X-ray under NICE guidelines that is, hemoptysis, or any of the following unexplained or persistent (that is, lasting more than 3 weeks) symptoms or signs: cough, chest/shoulder pain, dyspnea, weight loss, chest signs, hoarseness, finger clubbing, features suggestive of metastasis from a lung cancer (for example, in brain, bone, liver or skin), or cervical/supraclavicular lymphadenopathy; 2) patients who have had a chest X-ray or CT scan of the chest in the previous 3 months; 3) patients who need a chest X-ray within the following 3 weeks for reasons other than those listed above; or 4) patients previously diagnosed with cancer who have a life expectancy of less than 1 year.

### Sample size considerations

#### ***Estimating the prevalence of extra-NICE symptoms in patients consulting UK general practices***

We have modeled the study using the CAPER (Cancer Prediction in Exeter) lung database [12,15], which contains all primary care consultations, symptoms, and investigations in the 2 years before diagnosis for a 5-year cohort of 247 lung cancer cases, to estimate the potential benefit of expanded chest X-ray use. Smokers aged over 60 years make up 3.2% of the UK population, and we estimate that in a 1-year period, 24% of these would present with extra-NICE symptoms only; that is, 0.768% (or 42 per year in an average practice of 5,500 patients). Hence, if we were to recruit 26 practices (each with a patient population of 5,500) and recruited patients over an average of 6 months (a 12 month recruitment period with practices opening at different points throughout that period), we could get an estimate of this with confidence intervals of 0.70 to 0.83% from a patient-year population of 71,500 (26 × 5,500 × 0.5).

#### ***Estimating the proportion of those with extra-NICE symptoms who will agree to participate***

If we were to find a prevalence of extra-NICE symptoms of 0.768% per year then, from the above, we might find 550 patients eligible for the study. Studies conducted at the Wales Cancer Trials Unit have found a 70% consent rate of eligible patients into trials. We could get an estimate of this with confidence intervals of 70 to 74% in this study.

#### ***Estimating the proportion of those who agree to participate and who are diagnosed with lung cancer***

If 386 (70%) eligible patients consented to participate in the trial, then we could get 95% confidence intervals of 0.9 to 3.9% around an estimate of 2.4% (from CAPER lung database) of those who will be diagnosed with lung cancer.

It was decided to try to open all practices to recruitment for at least 12 months during the running of the trial, so as to avoid any selection bias caused by symptom seasonality.

### Method of randomization

Randomization will take place centrally at the Bristol Randomised Trials Collaboration using either an automated telephone or a secure online service. Participants will be randomized using minimization with a random element. This will ensure balanced treatment allocation by a number of clinically important factors: GP practice, age (<75 or ≥75 years), and COPD diagnosis. Randomization will have an allocation ratio of 1:1.

### Outcome measures and statistical analysis

The primary analysis will be intention to treat.

#### ***Primary outcome measures***

The primary outcome measures are as follows.

• The prevalence of extra-NICE symptoms in patients consulting a UK general practice. Participating practices will be asked to log a count of all eligible patients. The prevalence will be calculated as the total number of eligible patients logged by the participating practices divided by the total number of patients on their lists.

• The proportion of those who agree to participate. This will be known from trial recruitment, and calculated as the total number of patients recruited by the participating practices divided by the total number of eligible patients logged by them.

• The proportion of those that are diagnosed with lung cancer. This will be obtained from the UK National Lung Cancer Audit Database (LUCADA; available from the NHS Information Centre) and the general practices. It will be calculated as the total number of participating patients found to have lung cancer divided by the total number of patients recruited. A statistical comparison of the proportion in each arm will be made.

#### ***Secondary outcome measures***

We will also assess the following secondary outcome measures

• Of those patients diagnosed with lung cancer, we will assess stage at diagnosis, performance status, and the proportion of patients receiving radical treatments. This will be obtained from LUCADA and the general practices, and proportions will be presented overall and by trial arm.

• Anxiety/depression that may be caused by unnecessary or no chest X-rays. This will be measured using the Hospital Anxiety and Depression Scale (HADS) [32] and informed by the qualitative interviews. This will be presented overall and by trial arm.

• Assessment of false-positive (suspicion of cancer on X-ray but no subsequent cancer diagnosis within 6 months) and false-negative (no suspicion of cancer on the X-ray but diagnosis of lung cancer within 6 months) rates for the chest X-rays. This will be obtained from LUCADA and the general practices, and proportions will be presented overall and by trial arm.

• Cost-effectiveness of extra-NICE. This will be measured by a Client Service Receipt Inventory (CSRI), the ICECAP (O) (Investigating Choice Experiments for the Preferences of Older People (ICEPOP) Capability measure [33], and the EQ-5D-3 L (EuroQol 5 Dimensional Health State Questionnaire). The CSRI completed by the patient will be compared against data about resource use available from the GP to ascertain the best method of capturing these data in the full trial.

The following processes will be reviewed and refined during the workshop at the start of the trial, during qualitative interviews, and continuously throughout the trial by the trial management group (TMG): 1) the best way to train GPs to identify and recruit eligible patients into the trial; 2) the most effective method of presenting the trial (and randomization) to patients and 3) barriers to recruitment and how can we overcome them,

### Data collection

#### ***Screening***

The number of eligible patients and the number of those who consented will be recorded by each general practice on screening logs.

#### ***Baseline data***

Patient name, NHS number, Trial number, and their address will be collected to facilitate data linkage with routine datasets and administration of postal questionnaires (see below). This patient identifiable information will be sent by fax at the general practice to a secure fax at the Wales Cancer Trials Unit (WCTU) by the practice staff as soon as possible after the patient is randomized. The original consent form will then be stored in the Investigator Site File at the practice.

The following data will be collected on a Case Report Form (CRF) (using non-carbon copy paper) completed by staff at the general practice at the time of recruitment: trial number, patient initials, date of birth, gender, ethnicity, socioeconomic status, and educational level; co-morbidity (Charlson Index); venue for determination of eligibility; primary reason for presenting; ex-smoker or current smoker, number ofpack years, and the quit date for ex-smokers; presence of pre-existing lung disease; date of previous chest X-ray in the preceding 12 months; and presenting symptoms and duration.

The ICECAP(O), HADS, EQ-5D-3 L and CSRI will be given to the patient to complete at the time of randomization.

#### ***Two months after randomization***

The ICECAP (O), HADS, EQ-5D-3 L, and CSRI will be posted to the patient at home to complete and return in a stamped addressed envelope. Patients will also be invited to give open comments about their experience on the trial. Staff at WCTU will check first with the patient’s GP to ensure that they are still alive before sending the questionnaires to the patient’s home address. If the patient returns the completed questionnaires, then they will be sent a £5.00 voucher as thanks. A reminder letter will be sent if no response is received within 2 weeks, and a telephone call reminder will be made if there is no response within 4 weeks [34]. A score on the completed HADS questionnaires of either less than 16 for anxiety or less than 11 for depression will trigger letters indicating the high scores to both the patient and their GP.

#### ***During the trial***

General practices will be asked to append an additional sheet onto the chest X-ray request forms or letters for the patients randomized to either arm of the trial. This sheet will highlight that the patient is part of a trial, and will ask the radiology department to fax a copy of the chest X-ray result to the WCTU secure fax. In the case of missing forms, the GP practice will be contacted and asked to fax this to WCTU.

#### ***Twelve months after randomization***

LUCADA will be used to collect the following data by data linkage: date of diagnosis of lung cancer; lung cancer stage at diagnosis; date of resection; WHO performance status at diagnosis; and date and cause of death.

Additionally, 12 months after randomization is completed, general practices will be sent a list of the patients that they have enrolled into the trial and asked to return the following information to WCTU: 1) a completed CRF with the following data for those patients: health resource use because being enrolled into the trial; and follow-up of any abnormal/indeterminate chest X-rays (for example, any repeat X-rays performed, and results of any biopsies); 2) a print-out (from the practice computer system) of all consultations with GPs and nurses, all prescriptions, and all results of GP-initiated investigations.

### Health economics

This feasibility study offers the opportunity to explore the most research-efficient way of incorporating economic evaluation into the full trial [35]. Specifically, we will focus on piloting methods for collecting primary and secondary care resource use using routine data as compared with patient recall (which is unlikely to be satisfactory given the complexity of cancer treatment). We will also have the opportunity to pilot use of the EQ-5D-3 L and ICECAP (O) in this population group and diagnostic context. Although the EQ-5D-3 L instrument is the most widely used generic quality of life measure in economic evaluation studies for quality-adjusted life years calculation, it still requires methodological benchmarking against other health-related quality of life measures. Specifically there is growing interest in the ICECAP (O) measure, which is based on capability theory rather than the more functional aspects of health-related quality of life as in EQ-5D.

### Qualitative interviews

The qualitative component will explore: 1) GPs’ experiences of recruiting and consenting patients, including the time and logistical implications of these experiences; and 2) Patient response to recruitment and participation.

It is anticipated that the data from this component of the trial will be used to help identify: the best way to train primary care staff to identify and recruit eligible patients into the trial; the most effective method of presenting the trial (and randomization) to patients; and barriers to recruitment and potential solutions.

### Sampling strategy

Qualitative interviews will be conducted with four groups: a sample of GPs, patients randomized to extra-NICE, patients randomized to NICE, and, if possible, patients who refuse participation in the trial. We expect that interviews with 10 GPs and up to 30 patients from across the three groups will be sufficient to gain the required insight, and this is in line with the usual recommendations for sample sizes for a Framework analysis [36]. Participants will be purposively selected until the required number is recruited.

### Obtaining consent and approaching patients

Consent for the GP interviews will be sought at the time they agree to participate in the trial. All patients who are invited to participate in the trial will be asked at this time to additionally consent to be interviewed, although it will be explained that only a small number of patients will be approached for interview. Patients who refuse participation in the clinical trial will also be invited at this time to consent to an interview about their reasons for non-participation. A separate Participant Information Sheet and Consent Form for the qualitative interviews will be provided to trial participants. The researcher will receive from the participating general practices, the name, address, and telephone number of all participants who have consented to be interviewed. The research interviewers will contact participants who consent and arrange a convenient time and location for interview. as follows. Non-trial participants will be contacted as soon as possible after consent is obtained, patients in the trial arms will be contacted once consent and randomization has taken place, and GPs will be contacted once recruitment to the trial is underway.

The GP interviews will be split into three groups; low, medium, and high recruiters. Convenience sampling will be used to recruit participants to the interviews, that is, they will be contacted by the researcher following their clinical appointment with the GP and having given consent.

### The interviews

Individual participants will be interviewed at home or in a quiet clinic location, according to their preference. The interviews will be semi-structured according to an initial interview schedule. The schedule will be adjusted to account for patient-led topics, if necessary, following the first two or three interviews. This iterative process allows the patients’ topic(s) of interest to be heard and accounted for within the data collection. The interviewer will digitally record the interview, but will also make field notes (with the patient’s permission) to record incidents occurring during the interview, non-verbal communication, or reactions at the time of the interview. Interviews will be 30 to 60 minutes in length, and will be terminated earlier if the participant is thought to be fatigued or becomes unwell. Ideally, patients will be interviewed alone but, if they prefer, a friend or relative may be present.

### Data transfer and transcription

The interviewer will upload the digital media files onto a secure computer, and files will be labeled with a study number. No identifiable data will be stored. Digital files will be stored at the WCTU. The transcription secretary at the WCTU will transcribe the interviews verbatim, and following the WCTU transcription, will use standard operating procedures to ensure data protection and confidentiality. The transcripts will be uploaded to the NVivo 8 qualitative software program for efficient data management.

### Analytic framework

Data analysis will be performed using a Framework approach [36]. This is a pragmatic approach to qualitative data analysis that emerged from policy research.

The quality and veracity of the analysis will be checked throughout. For example, if during analysis, the researcher is uncertain of the meaning or interpretation of a section of interview data, the participant will be contacted for verification. In addition, double coding and analysis will be conducted by the qualitative lead. Ongoing supervision and discussion with senior researchers and the trial team will support the reflexivity of the researcher and reflection on their interpretation in order to ensure findings are grounded in the data.

### Presentation of results

The anonymized data will be represented by selected extracts in a narrative format with a thematic structure. The results will be discussed with data extracts used in support of claims made. The TMG will review the results to assess potential alterations to trial design, and will include the qualitative analysis to complement the reporting of the full trial, where appropriate.

## Discussion

This trial will assess the feasibility and inform the design of a large, UK-wide, clinical trial of a change to the NICE guidelines for urgent referral for chest X-ray for suspected lung cancer. It utilizes a combination of workshop, health economics, quality of life, qualitative and quantitative methods in order to fully assess feasibility.

This study has been designed as an individually randomized study. The design team debated the merits of cluster randomization (with practices being randomized to either Arm A or Arm B) to ease the administrative burden associated with recruiting patients and to prevent potential contamination by patients randomized to Arm A potentially seeking chest X-rays elsewhere. However, cluster randomization may have created selection bias with more patients potentially being recruited into Arm A practices. Additionally, cluster randomization requires more patients in power calculations for comparisons of primary endpoints (to account for the clustering), which would make a future definitive trial more difficult to achieve.

## Trial status

At the time of manuscript submission, this trial was still in the patient recruitment phase.

## Competing interests

WH is the clinical lead for the current revision of the NICE 2005 guidance. His contribution to this article is in a personal capacity, and is not to be interpreted as representing the view of the Guideline Development Group, or of NICE itself. None of the other authors has any competing interests.

## Authors’ contributions

TKR and RDN were responsible for the research question; CNH is responsible for the design, sample size calculations, and statistical analysis of the study; RDN, TKR, and WH provided clinical expertise for the design; KH and GOG provided expert advice on the study design as clinical triallists; KR is the trial manager for the study; AN and AT were responsible for the design of the qualitative component; HP is conducting the qualitative interviews and analysis; RTE was responsible for the design of the health economic component of the trial; STY is responsible for analyzing the health economics data; JF and AB are the patient representatives on the study; ETJ has advised on trial management and feasibility of trial set-up in primary care; and CH and RDN prepared the first draft of this manuscript. All authors contributed to the writing of the protocol and have read and approved the final manuscript. RDN and GOG will act as guarantors.
